# Transcriptomic differences between fibrotic and non-fibrotic testicular tissue reveal possible key players in Klinefelter syndrome-related testicular fibrosis

**DOI:** 10.1038/s41598-022-26011-6

**Published:** 2022-12-13

**Authors:** Margo Willems, Catharina Olsen, Ben Caljon, Veerle Vloeberghs, Jean De Schepper, Herman Tournaye, Dorien Van Saen, Ellen Goossens

**Affiliations:** 1 Laboratory for Reproduction, Genetics and Regenerative Medicine, Department of Reproduction, Genetics and Regenerative Medicine, Biology of the Testis (BITE) Laboratory, Vrije Universiteit Brussel (VUB), Laarbeeklaan 103, 1090 Brussels, Belgium; 2 Brussels Interuniversity Genomics High Throughput Core (BRIGHTcore) Platform, Universitair Ziekenhuis Brussel (UZ Brussel)/Vrije Universiteit Brussel (VUB), Laarbeeklaan 101, 1090 Brussels, Belgium; 3 Universitair Ziekenhuis Brussel (UZ Brussel), Brussels IVF, Laarbeeklaan 101, 1090 Brussels, Belgium; 4 Department of Pediatrics, Division of Pediatric Endocrinology, Universitair Ziekenhuis Brussel (UZ Brussel), Laarbeeklaan 101, 1090 Brussels, Belgium; 5 Department of Obstetrics, Gynecology, Perinatology and Reproduction, Institute of Professional Education, Sechenov First Moscow State Medical University of the Ministry of Health of the Russian Federation, Trubetskaya Str. 8, B. 2, 119992 Moscow, Russia

**Keywords:** Sequencing, Infertility, Inflammation

## Abstract

Klinefelter syndrome (KS; 47,XXY) affects 1–2 in 1000 males. Most men with KS suffer from an early germ cell loss and testicular fibrosis from puberty onwards. Mechanisms responsible for these processes remain unknown. Previous genomics studies on testis tissue from men with KS focused on germ cell loss, while a transcriptomic analysis focused on testicular fibrosis has not yet been performed. This study aimed to identify factors involved in the fibrotic remodelling of KS testes by analysing the transcriptome of fibrotic and non-fibrotic testicular tissue. RNA sequencing was performed to compare the genes expressed in testicular samples with (KS and testis atrophy) and without (Sertoli cell-only syndrome and fertile controls) fibrosis (n = 5, each). Additionally, differentially expressed genes (DEGs) between KS and testis atrophy samples were studied to reveal KS-specific fibrotic genes. DEGs were considered significant when p < 0.01 and log2FC > 2. Next, downstream analyses (GO and KEGG) were performed. Lastly, RNA in situ hybridization was performed to validate the results. The first analysis (fibrotic vs non-fibrotic) resulted in 734 significant DEGs (167 up- and 567 down-regulated). Genes involved in the extracellular structure organization (e.g. *VCAM1*) were found up-regulated. KEGG analysis showed an up-regulation of genes involved in the TGF-β pathway. The KS vs testis atrophy analysis resulted in 539 significant DEGs (59 up- and 480 down-regulated). Chronic inflammatory response genes were found up-regulated. The overlap of X-linked DEGs from the two analyses revealed three genes: matrix-remodelling associated 5 (*MXRA5*), doublecortin (*DCX*) and variable charge X-Linked 3B (*VCX3B*). RNA in situ hybridization showed an overexpression of *VCAM1*, *MXRA5* and *DCX* within the fibrotic group compared with the non-fibrotic group. To summarize, this study revealed DEGs between fibrotic and non-fibrotic testis tissue, including *VCAM1*. In addition, X-linked fibrotic genes were revealed, e.g. *MXRA5, DCX* and *VCX3B*. Their potential role in KS-related testicular fibrosis needs further study.

## Introduction

Klinefelter syndrome (KS) was first documented in 1942 and remains the most common sex-chromosome related disorder to this day with KS being diagnosed in approximately 1 to 2 in 1000 new-born males^1–3^. Men diagnosed with KS show at least one supplementary X-chromosome (47,XXY) and may present some KS features including small testes, azoospermia and hypogonadism. Due to the absence of any significant symptoms or features in many patients, this disorder remains highly underdiagnosed, with only a presumable 25% of all patients actually being diagnosed^4–7^. Diagnosis mainly occurs during adulthood when men with KS discover they are unable to conceive naturally, since azoospermia can be seen in more than 90% of these patients. Germ cell loss is already noticeable during childhood and has even been shown during fetal life^8–10^. The loss of germ cells has been suggested to be a consequence of either the chromosomally aberrant germ cells present in KS testes, a disturbed testicular environment or the altered gene dosage^11^. Fertility strategies in adult KS patients include a testicular sperm extraction (TESE) procedure followed by an intracytoplasmic sperm injection. However, the TESE procedure only appears to be successful in about 48% of adult KS patients^12,13^. The success-rate of finding spermatozoa/spermatogonia is hampered because of testicular fibrosis, which occurs from puberty onwards, and which eventually leads to the degeneration of the seminiferous tubules within the KS testicular tissue^8,9^. Unfortunately, the exact mechanisms responsible for the initiation of testicular fibrosis in men with KS are still unknown.

Several studies on the transcriptomic analysis of KS samples have been reported. In five of these studies, RNA was isolated from whole blood to compare the transcriptome of KS patients to controls^14–18^. Vawter et al. specifically studied the genes associated with verbal cognition whereas the research of Huang et al. was more focussed on the relation between the gene expression and the regulation of metabolism in KS patients compared to controls^17,18^. Other transcriptomic analyses were performed to unravel which genes are responsible for the typical KS phenotype, revealing differentially expressed genes (DEGs) involved in multiple pathways, including, among others, insulin resistance, male infertility, azoospermia and obesity^14–16^. D’Aurora et al. performed microarray transcriptome analyses on fresh testicular biopsies from azoospermic KS patients^19^ and testicular biopsies from KS patients with ‘hypospermatogenesis’^20^ to uncover DEGs related to germ cell loss. These analyses revealed DEGs involved in steroidogenesis, spermatogenesis failure, germ cell function and morphology, cell death and blood-testis barrier maintenance. Winge et al. performed a transcriptome analysis on fetal KS and age-matched controls to find an answer on how the supplemental X-chromosome affects adult spermatogenesis. The results from this research showed changes in the germ cell differentiation pattern and DEGs involved in somatic cell maturation, hormone response, cell death and testis development^10^. In addition, transcriptomic analysis on the adult human KS paraffin-embedded testis was performed to uncover DEGs involved in germ cell loss and the destruction of seminiferous tubules. This analysis showed the disturbed differentiation of the somatic cell populations in the KS testes and revealed that transcriptomes of testes from different developmental ages show limited overlap^21^. Single cell RNA sequencing studies have also already been conducted on KS testis tissue. Laurentino et al*.* studied the epigenetic and transcriptional profiles of KS germ cells. This study included one KS patient and showed a normal transcriptome but imprinting aberrations for the KS germ cells^22^. Zhao et al*.* included seven men with non-obstructive azoospermia (NOA) in their study, of which three were KS patients, and compared them to ten healthy donors of different age categories to gain insights in the NOA pathogenesis. The analysis showed the biggest difference of transcriptomic profile in the Sertoli cell population between NOA patients and controls. KS Sertoli cells expressed the highest proportion of X-linked genes, which suggests that this makes KS Sertoli cells more susceptible to dysregulation due to the supplemental X chromosome^23^. The single cell sequencing study by Mahyari et al*.* also revealed a central role for Sertoli cells in the pathology of KS patients, as the Sertoli cells seem to lose their X-inactivation during puberty^24^.

Since most of these studies were focussed on the DEGs involved in germ cell loss, the role of genes which may be involved in the testicular fibrotic process were not specifically investigated. Nevertheless, the inflammation pathway was uncovered as one of the associated processes in three of the studies^14,19,20^. In addition, the study by Mahyari et al*.* revealed a couple of genes related to testicular fibrosis^24^.

The aim of the current study was to reveal DEGs which are involved in the fibrotic process in KS. Unravelling the mechanisms behind the testicular fibrosis in KS patients could uncover how to delay or even stop the fibrotic process, leading to a higher chance of sperm retrieval in KS patients through a TESE procedure. Therefore, RNA sequencing was performed on fresh testicular biopsies from adult KS patients and patients with mixed testis atrophy (TA), who show testicular fibrosis and in whom germ cells can be present (fibrotic group), Sertoli cell only syndrome (SCO) patients, who lost the germ cells but do not show any fibrosis in their testes and fertile men (non-fibrotic group).

## Methods

### Patient samples

Testicular biopsy samples were obtained from adult KS (n = 5), SCO (n = 5) and TA patients (n = 5) during a TESE procedure in function of their fertility treatment. In addition, samples from men who underwent a vasectomy reversal (n = 5) were included as fertile controls (FC). Written informed consent was obtained from all patients included in this study. Patient samples were divided over four categories: non-fibrotic samples containing spermatozoa (FC), non-fibrotic samples lacking germ cells (SCO), samples with a normal karyotype showing testicular fibrosis (TA) and samples with the KS karyotype lacking germ cells and showing fibrosis (KS). More information about the patients included in this study can be found in Table 1.

**Table 1:** Individual information on patients included.

Patient ID	Reason for TESE procedure	Diagnosis/anamnesis	Age at biopsy	Karyotype	Fibrotic score	% tubules with germ cells (number)	% tubules with only sertoli cells (number)	% hyalinized tubules (number)	% interstitium
KS1	Non-obstructive azoospermia	Klinefelter syndrome	36	47, XXY	3	31 (4)	31 (4)	38 (5)	94
KS2	Non-obstructive azoospermia	Klinefelter syndrome	29	47, XXY	3	0	0	100 (58)	24
KS3	Non-obstructive azoospermia	Klinefelter syndrome	47	47, XXY	4	0	100 (2)	0	100
KS4	Non-obstructive azoospermia	Klinefelter syndrome	30	47, XXY	4	0	0	0	100
KS5	Non-obstructive azoospermia	Klinefelter syndrome	43	47, XXY	4	0	33 (5)	67 (10)	85
TA1	Non-obstructive azoospermia	Ideopathic testicular atrophy	37	46, XY	2	32 (12)	3 (1)	66 (25)	48
TA2	Non-obstructive azoospermia	Ideopathic testicular atrophy/cryptorchidy	35	46, XY	2	25 (8)	59 (19)	16 (5)	42
TA3	Non-obstructive azoospermia	Ideopathic testicular atrophy	38	46, XY	3	9 (2)	43 (9)	48 (10)	72
TA4	Non-obstructive azoospermia	Ideopathic testicular atrophy	29	46, XY	3	47 (28)	37 (22)	6 (10)	62
TA5	Non-obstructive azoospermia	Ideopathic testicular atrophy	49	46, XY	3	0	0	100 (66)	78
SCO1	Non-obstructive azoospermia	Sertoli cell only syndrome	31	46, XY	1	0	100 (43)	0	44
SCO2	Non-obstructive azoospermia	Sertoli cell only syndrome	29	46, XY	2	7 (3)	93 (38)	0	51
SCO3	Non-obstructive azoospermia	Sertoli cell only syndrome	35	46, XY	2	0	100 (77)	0	46
SCO4	Non-obstructive azoospermia	Sertoli cell only syndrome	34	46, XY	1	0	100 (81)	0	36
SCO5	Non-obstructive azoospermia	Sertoli cell only syndrome	37	46, XY	2	0	100 (36)	0	42
FC1	Vasectomy reversal	Vasectomy	52	46, XY	1	97 (35)	3 (1)	0	39
FC2	Vasectomy reversal	Vasectomy	49	46, XY	1	100 (58)	0	0	23
FC3	Vasectomy reversal	Vasectomy	50	46, XY	2	94 (49)	6 (3)	0	37
FC4	Vasectomy reversal	Vasectomy	39	46, XY	1	90 (37)	10 (4)	0	32
FC5	Vasectomy reversal	Vasectomy	37	46, XY	1	100 (41)	0	0	22

Immediately after the biopsy was retrieved, samples were snap frozen in liquid nitrogen after which they were stored at − 80 °C for next generation sequencing. In addition, biopsy samples were fixed in alcohol-formalin acetic acid mixture (AFA; Q02022; international medical products, Oudergem, Belgium) for at least one hour and further dehydrated and fixed with TISSUE TEK-VIP (5990; Sakura, Berchem, Belgium) overnight after which the tissue was embedded in paraffin. For validation by RNA in situ hybridization, sections of 5 mm thick were cut at three different depths using a microtome (SM2010R; Leica, Brussels, Belgium).

### Histological examination

The fibrotic score of each testicular tissue sample included in this study was determined as previously described^8^. In brief, paraffin-embedded tissue was used to perform a haematoxylin-periodic acid Schiff’s reagent (H/PAS) staining. The histology of the tissue was scored according to the degree of fibrosis. A mean score of five evaluated image fields was given to each tissue sample: (0) normal histology; (1) normal tubules but interstitial fibrosis; (2) degenerated tubules and interstitial fibrosis; (3) hyalinized tubules and severe fibrosis; (4) severe fibrosis, complete loss of tubules. If the mean score was not a round number, it was rounded up to the nearest round number. Only samples with a fibrotic score ≤ 2 were included in the non-fibrotic groups while for the fibrotic groups, only samples with a score of 3 or 4 were included for KS patients and samples with a score of 2–4 for the TA patients were included. Supplementary Fig. 1 shows the H/PAS staining of every testicular tissue piece included in this study.

To examinate the histology of the included testicular tissue more thoroughly, the percentage of tubules with ongoing spermatogenesis, tubules with an SCO image and hyalinized tubules was calculated. An immunohistochemical staining against melanoma associated antigen 4 (provided by Giulio Spagnoli, University of Basel, Switzerland) was performed to evaluate the presence of spermatogonial stem cells and spermatogonia as described before^8^. The number of tubules, as well as the area of interstitium, was evaluated in three tissue pieces at three different depts from every patient included, using the Fiji software^25^. The results of this analysis can be found in Table 1.

### RNAseq: Wetlab protocol

Total RNA was extracted from the testicular tissue using the RNeasy Micro kit (74004; Qiagen, Antwerp, Belgium) following the manufacturer’s guidelines. RNA concentration was determined with a Nanodrop spectrophotometer (Isogen Life Science, De Meern, the Netherlands). The subsequent steps of the RNAseq procedure were carried out at the BrightCore facility (UZ Brussel). Extracted RNA from KS, SCO, TA and FC biopsies was analysed on the Agilent 2100 Bioanalyzer system (G2939BA; Agilent technologies), leading to an RNA integrity number (RIN) score. Samples with RIN scores above 4.7 were considered pure enough to continue with the experiments. An overview of the RIN scores for the testicular tissue included in this study is shown in Aupplementary Table I. Subsequently, an input of 150 ng RNA per sample was processed using the KAPA RNA HyperPrep with RiboErase (08098131702; Roche, Vilvoorde, Belgium) library preparation kit with the following steps: oligo hybridization and RNA depletion, 2.2 × bead-based RNA depletion clean-up, DNase digestion, 2.2 × bead-based DNase digestion clean-up, RNA elution, fragmentation and priming, first strand synthesis, second strand synthesis and A-tailing, adaptor ligation, first 0.63 × bead-based post-ligation clean-up, second 0.7 × bead-based post-ligation clean-up, library amplification and library amplification clean-up. These steps were carried out according to the manufacturer’s protocol. Library validation was performed through the measurement of cDNA fragment concentrations, using the Victor Nivo Multimode Microplate Reader (HH35000500; PerkinElmer, Zaventem, Belgium), and the length of the different fragments was measured by the Fragment-analyzer (M5311AA; Agilent technologies). These steps were carried out according to the manufacturer’s protocol in order to obtain a library insert size of 300–400 bp.

Libraries were pooled according to the two different patient groups (fibrotic vs non-fibrotic tissue) as well as pooled within the fibrotic group (KS vs TA). The pooled libraries were sequenced with the Illumina NovaSeq 6000 (Illumina, Eindhoven, the Netherlands). The sequencing stats (coverage and percentage of aligned reads) are shown in Supplementary Table I. In addition, the sequencing are publicly available in the GEO database (GSE200680).

### RNAseq: differential expression and downstream analysis

The reads were mapped to the human genome (hg19) using the tool STAR v2.5.0c^26^. The mapped reads were then translated into a quantitative measure of gene expression, counting the number of reads that map to each exon/gene using HTSeq v0.11.0^27^.

Following quantification of expression levels, the differential expression between the different groups was determined. Focus was put on the protein coding genes (19,265 genes, downloaded from Ensemble biomart in May 2019). In a first step, a PCA analysis was run to check for outliers (Supplementary Fig. 2). Next, the differential expression analysis was run after which the genes that were significantly up- or down- regulated with an absolute log2 fold change > 2 after shrinkage (type *apeglm*) and an adjusted p-value < 0.01 were extracted. This was done with the *DESeq2* package v1.24.0 in R v3.6.1^28^. The correction of multiple testing was done with the Benjamini–Hochberg FDR implementation in DESeq2.

Then, two gene ontology (GO) analyses were run using (i) the set of up-regulated genes and (ii) the set of down-regulated genes using the *enrichGO* function of the Bioconductor package *clusterProfiler*. On these two gene sets, a kyoto encyclopedia of genes and genomes (KEGG) analysis was run using the *enrichKEGG* function of the same package^29^.

### RNA in situ hybridization

RNA in situ hybridization was used as validation method to confirm the overexpression of *VCAM1, DCX* and *MXRA5* in the fibrotic tissue. Two samples of one patient per diagnosis category (KS3, TA4, FC5, SCO1) were included for the validation. This was performed by the ‘extended RNA analysis’ facility at VUB (https://lmmo.research.vub.be/en/era-facility). The RNAscope 2.5 HD Duplex Assay (322430; Advanced Cell Diagnostics, Newark, CA, USA) was used according to the manufacturer's instructions with adapted pretreatment conditions (15 min target retrieval and 15 min protease plus). Probes used were B. subtilis duplex negative control (320751; Advanced Cell Diagnostics), H. sapiens duplex positive control (321641; Advanced Cell Diagnostics) and H. sapiens *MXRA5* (419691; Advanced Cell Diagnostics), *VCAM1* (440371-C2; Advanced Cell Diagnostics) and *DCX* (489551; Advanced Cell Diagnostics). Imaging was performed with the Aperio slide scanner (GT 450; Leica). Quantification of the RNA in situ hybridization was performed with the HALO software (Indicalabs)^30^. Two testicular tissue pieces from one patient of each diagnosis group (KS3, SCO1, FC5, TA1) were included for the quantification. A mean percentage of cells expressing the genes of interest (*VCAM1*, *MXRA5* and *DCX*) was calculated.

### Statistical analysis

The age of the patients in each group was compared by an unpaired t-test (GraphPad Prism8 software). The difference was considered statistically significant if p < 0.05.

### Ethics approval

The study was approved by the ethical committee of the UZ Brussel (2015/121). All methods were performed in accordance with the relevant guidelines and regulations.

## Results

### Transcriptomic analysis: fibrotic vs non-fibrotic tissue

No significant difference (p = 0.568) was found between the fibrotic and non-fibrotic group concerning age at biopsy (Supplementary Fig. 3).

**Figure 1:**
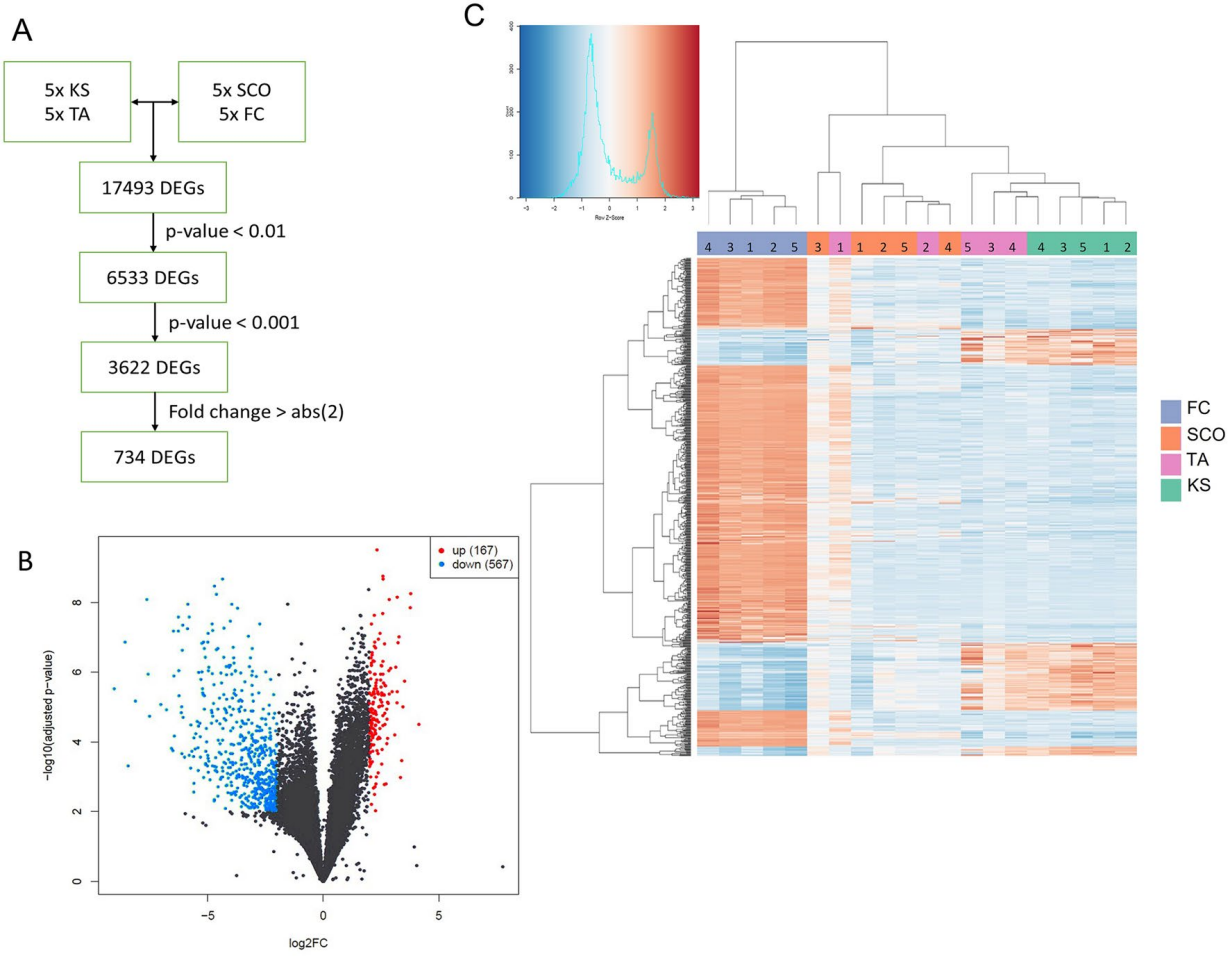
Gene expression analysis comparing fibrotic and non-fibrotic testis tissue. (**A**) Flowchart of the RNA sequencing data analysis, resulting in 734 differently expressed genes (DEGs). (**B**) Volcano plot of the genes found by RNA sequencing analysis revealed 167 up- and 567 down-regulated significant DEGs in the fibrotic group vs the non-fibrotic group. (**C**) Heatmap of the DEGs for comparing Klinefelter syndrome (KS) and mixed testis atrophy (TA) samples with Sertoli cell only syndrome (SCO) and fertile control (FC) samples. KS and TA samples with the highest fibrotic scores cluster together.

**Figure 2:**
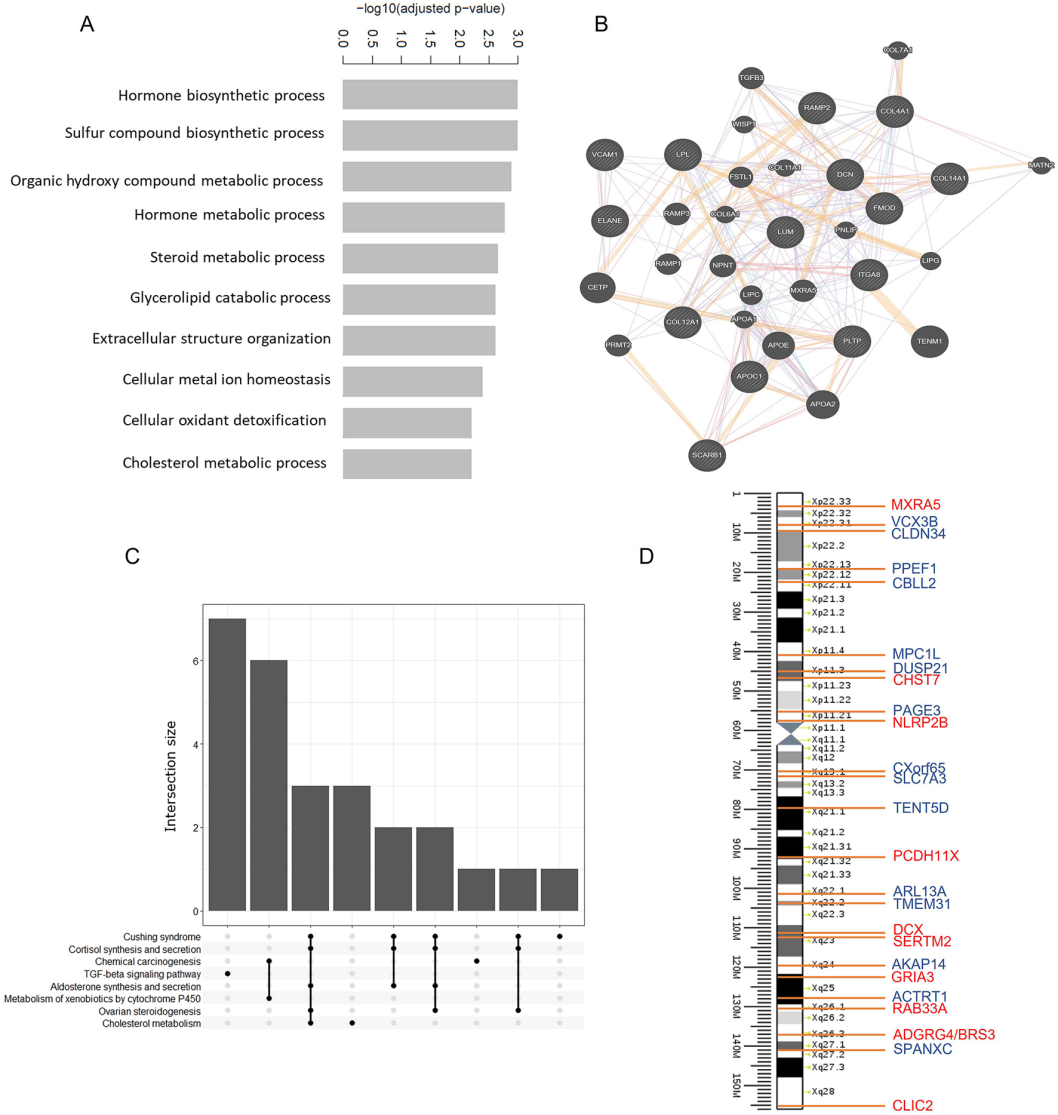
Enrichment analysis of the fibrotic versus non-fibrotic analysis. (**A**) Gene ontology analysis of the biological functions of the up-regulated genes (**B**) Extracellular structure organization network (rendered by Genemania). (**C**) Result of the KEGG analysis of the up-regulated genes, showing the 8 highest expressed pathways. In addition, the intersection size (e.g. number of overlapping genes) between the top pathways is shown. (**D**) All significant X-linked DEGs: 11 genes were up-regulated (red) and 15 genes were down-regulated (blue).

Transcriptomic analysis resulted in a total of 734 significant DEGs, of which 167 were up-regulated and 567 were down-regulated in the fibrotic group (KS + TA) in comparison to the non-fibrotic group (SCO + FC) (Fig. 1A,B; Supplementary Information 1—Significant DEGs). Results showed that all FC samples clustered together, as did the KS samples, while the gene expression in SCO and TA samples was more diverse. Nevertheless, the TA samples with a fibrotic score of 3 clustered together with the KS samples (Fig. 1C). GO analysis showed diverse biological functions for the up-regulated DEGs. The top ten of the most up-regulated functions is shown in Fig. 2A. The most interesting function found through this GO analysis included the extracellular structure organization, since testicular fibrosis can be attributed to an excessive deposition of ECM components. The genes involved the extracellular structure organization, were, among others, decorin, lumican, vascular cell adhesion molecule 1 (*VCAM1*) and several collagen-related genes. The network of genes involved in this biological function is displayed in Fig. 2B. GO analysis for the down-regulated genes resulted in biological functions which are mainly related to spermatogenesis and fertilization, such as spermatid development and differentiation (Supplementary Fig. 4). KEGG analysis showed the up-regulation of genes involved in different pathways. Figure 2C shows the 8 most up-regulated pathways and their intersection size, meaning the number of up-regulated genes which are involved in these pathways, separately or overlapping. An up-regulation of genes involved in the transforming growth factor beta-1 (TGF-β1) pathway was found. The up-regulation of *TGF-β1* in KS patients compared with SCO and TA patients and fertile controls has been validated by RT-qPCR analysis and was found significant (Supplementary Information 2 and Supplementary Fig. 5). Moreover, a down-regulation of genes involved in the extracellular matrix (ECM)-receptor interaction pathway was seen. A total of 15 down-regulated and 11 up-regulated DEGs were located on the X-chromosome (Fig. 2D). An overview of the number of DEGs per chromosome is shown in Supplementary Fig. 6A.

### Transcriptomic analysis—KS vs TA

**Figure 3:**
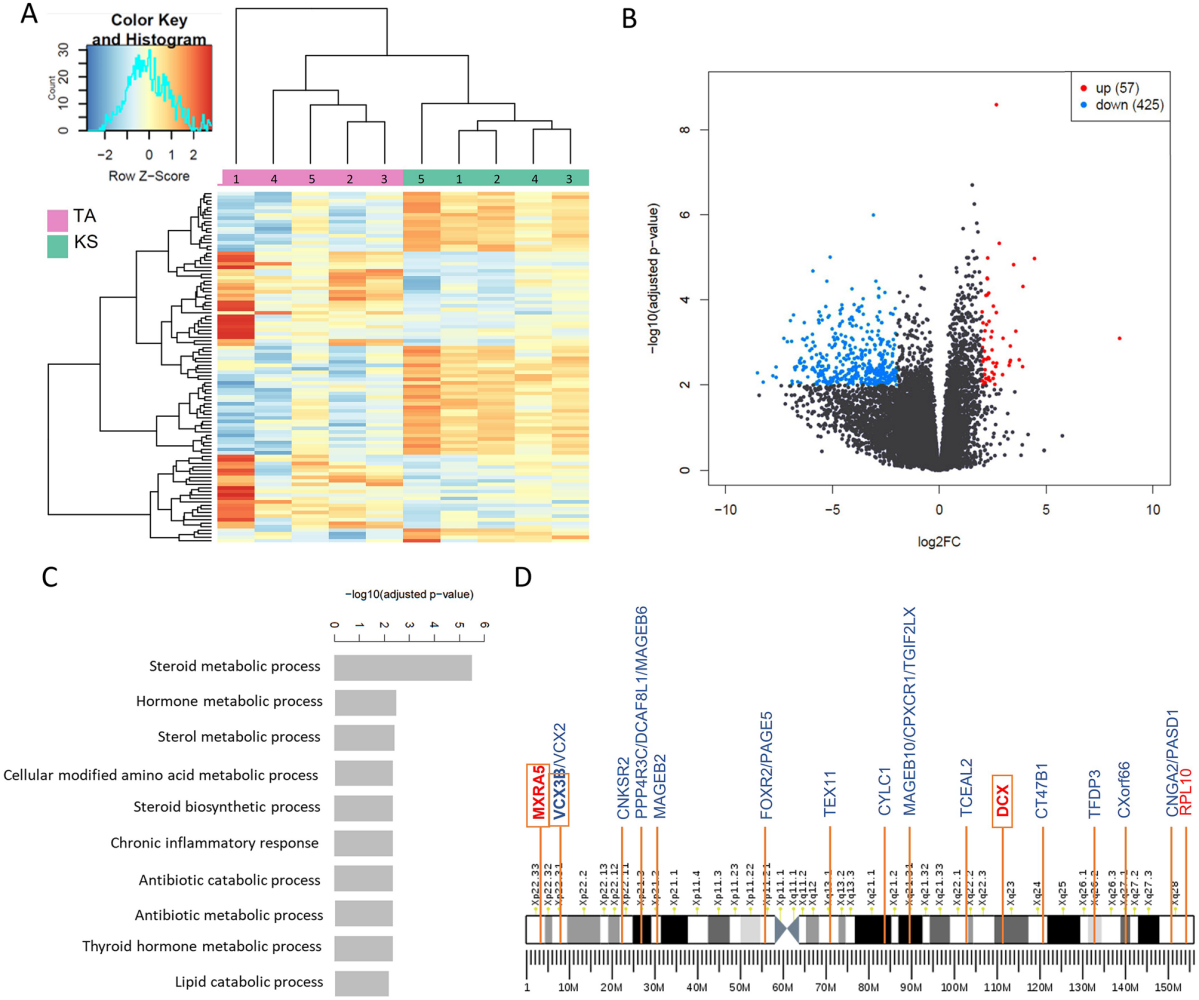
Gene expression analysis comparing KS and TA samples. (**A**) Heatmap of the data analysis, comparing TA and KS gene expression. (**B**) Volcano plot of the genes found by RNA sequencing analysis revealed 57 significantly up- and 425 down-regulated DEGs in the KS samples compared to the TA samples. (**C**) Gene ontology of the biological functions of the up-regulated genes. (**D**) All significantly X-linked DEGs are shown: three genes were up-regulated (red) and 21 were down-regulated (blue). Genes identified in both gene expression analyses are shown in an orange box.

Next, a transcriptomic analysis to find DEGs between the two fibrotic groups, KS and TA samples, was performed. The heatmap showed a clustering of KS samples and a clustering of TA samples (Fig. 3A). A total of 482 significant DEGs were found, of which 57 were up-regulated and 425 were down-regulated in the KS samples compared to the TA samples (Fig. 3B). Enrichment analysis revealed the most significant biological functions for both the up- and down-regulated genes. Among the up-regulated biological functions, the chronic inflammatory response (*PTGES/GJA1/CYP19A1*) was found (Fig. 3C). Processes involved in cilium movement, meiotic cell cycle and the cellular process involved in reproduction in multicellular organisms were found as most down-regulated biological functions (Supplementary Fig. 7). KEGG analysis for the up-regulated genes showed the steroid hormone biosynthesis as most up-regulated pathway. For the down-regulated genes, several pathways were found, including ABC transporters, tight junction and glycosaminoglycan degradation (Supplementary Fig. 8).

Twenty-one down-regulated genes were located on the X-chromosome, while three up-regulated DEGs could be found on the X-chromosome (Fig. 3D). An overview of the number of DEGs per chromosome for the second analysis is shown in Supplementary Fig. 6B. Concerning the X-linked genes, three genes were found in both the first analysis, comparing fibrotic and non-fibrotic tissue, and the second analysis, comparing fibrotic tissue with and without a normal karyotype: up-regulated matrix-remodelling associated 5 (*MXRA5*), up-regulated doublecortin (*DCX*) and down-regulated variable charge X-Linked 3B (*VCX3B*).

### Validation

**Figure 4:**
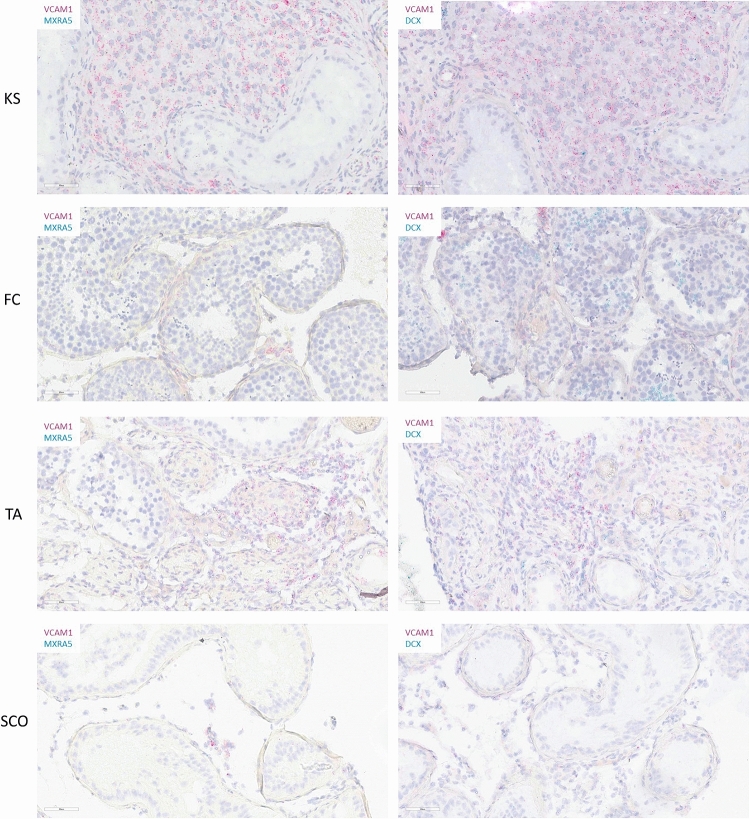
Validation of the gene expression results by RNA in situ hybridization. Duplex analysis showing a higher expression for all genes of interest (*VCAM1*, *MXRA5* and *DCX*) in KS samples compared with the control samples.

RNA in situ hybridization was performed to visualize three selected genes from our two analyses, namely *VCAM1*, *MXRA5* and *DCX*. *VCAM1* was selected due to its role in the inflammation pathway, while *MXRA5* and *DCX* were chosen as the two up-regulated X linked genes which overlapped in the two analyses of this study. The duplex array combined *VCAM1* with *MXRA5* and with *DCX*. In both combinations, an overexpression of *VCAM1* was clearly observed in KS samples compared with FC samples. A total of 84% of cells within the KS samples expressed *VCAM1* while this expression was only seen in 17% of all cells in the FC samples. *VCAM1* RNA molecules were mainly present in the interstitial tissue in FC and SCO samples, while in KS samples, *VCAM1* expression seems to correlate with Leydig cell hyperplasia. *VCAM1* expression was detected in 36% and 16% of all cells in the TA and SCO samples, respectively. *MXRA5* expression was barely seen in FC (7% of cells) and SCO (2% of cells) samples, while visibly more expression was detected in KS (41%) and TA (14%) samples. Quite some *DCX* RNA molecules could be distinguished in KS (28%) and TA (16%) samples, in contrast to SCO (4%) and FC (7%) samples. In FC samples, some positive patches were seen in the centre of the seminiferous tubules, however, this was also visible in the negative control of all FC samples included, confirming these patches did not correspond with real *DCX* expression (Fig. 4 and Supplementary Fig. 9).

## Discussion

The exact association between the supplementary X chromosome and the pathophysiology seen in men with KS has not yet been elucidated. In this study, a differential gene expression analysis was performed on KS and control testicular tissue to reveal a number of genes which may be responsible for the KS-related testicular fibrosis. First, a gene expression analysis of fibrotic versus non-fibrotic testis tissue was performed. In aging men, testicular fibrosis occurs naturally^31^, however, a statistical analysis showed no significant differences between the two patient groups in their respective age periods. The most down-regulated biological functions were associated with fertilization and spermatogenesis. For the up-regulated biological functions, both processes involving hormonal changes and processes of extracellular structure organization were found. Testicular fibrosis is characterized by excessive ECM synthesis and it has been shown that the tubular wall is remodelled in men with impaired spermatogenesis^32^. A recent study of our group revealed significant changes in the ECM of KS compared to SCO and FC, i.e. a loss of alpha smooth muscle actin 2 and an altered expression of collagen I and IV^33^. Several genes are involved within the ECM organization, including some collagen genes, VCAM1 and small leucine-rich proteins such as decorin and lumican. Decorin has already been shown to be highly expressed in testis tissue from men with KS, especially in hyalinized tubules^34^. Loss of lumican has been shown to ameliorate cardiac fibrosis^35^. *VCAM1* is expressed by the Leydig cells and can also be found in the basal parts of the Sertoli cells and is known to play a role in the testicular immunoregulation^36,37^. Recently, *VCAM1* has been shown to be crucial for the reconstruction of testicular interstitial tissue in vitro^38^. In addition, *VCAM1* is a key element of the inflammation pathway, contributing to several immunological disorders as well as cancer. Furthermore, *VCAM1* has already been associated with pulmonary fibrosis^39,40^. Our results also showed that DEGs involved in the TGF-β1 pathway were significantly up-regulated. It is known that testicular fibrosis is mediated by *TGF-β1* and that its expression is increased in men with impaired spermatogenesis^41,42^. Moreover, TGF-β1 production is regulated by decorin and vice versa^43^. In addition, it has been shown that *VCAM1* is a *TGF-β1* inducible gene^39^. In accordance, our research also revealed a significantly higher expression of *TGF-β1* in KS biopsies compared with fertile controls. Recently, a study from Mahyari et al. revealed different up-regulated genes related to fibrosis, specifically expressed by Leydig cells, through a single-cell analysis of KS biopsies^24^. Three of these profibrotic genes (*IGFBP5/WFIKKN2/ INHBA*) were also found up-regulated in our data.

Secondly, a gene expression analysis between the two fibrotic groups, KS and TA, was performed. This analysis, in combination with the first analysis, revealed an overlap of three genes: *MXRA5, DCX* and *VCX3B. MXRA5* is a proteoglycan which has a function in cell adhesion and ECM remodelling. *MXRA5* has been associated with several diseases and conditions, including preeclampsia and colorectal cancer^44,45^. A few years ago, *MXRA5* was identified as an anti-inflammatory and anti-fibrotic molecule, regulated by *TGF-β1*^46^. However, no exact function of *MXRA5* within the testis has been elucidated. *DCX* has been elaborately studied within the brain, as it is a microtubule-associated protein required for cerebral cortex development. In addition, it has been correlated with several disorders of the neuronal structures, including lissencephaly, epilepsy and developmental dyslexia^47^. A novel human *DCX* domain containing gene which is expressed mainly in the adult testis was discovered several years ago, however, the role of this protein within the testis is unknown^48^. *VCX3B* is a member of the *VCX/Y* gene family, of which all the members are exclusively expressed in male germ cells. The exact function of these genes is currently unknown, although the deletion of one of the family members, *VCX-A*, has already been associated with mental retardation, a condition also found in some patients with KS^49,50^. The single cell data set from the KS tissue included in Laurentino et al. showed expression of *MXRA5* in PTMCs, expression of *DCX* in Leydig cells and PTMCs and expression of VCX3B in germ cells^22^. Moreover, the KEGG analysis revealed genes involved in steroidogenesis pathogenesis to be up-regulated in the KS samples, which is in accordance with research showing an increased intratesticular testosterone level in men with KS^51^. Although no changes in the blood vessel density were found in KS testicular biopsies compared to controls, as reported in our previous research^34^, the above finding may contribute to the hypothesis that a disturbed vascularization is present within the KS testis^52,53^. In men with KS, as in females, one of the two X-chromosomes is silenced to compensate for the dosage of X-linked genes. However, it is known that about 15% of these genes escape X-inactivation, resulting in an overexpression of several X-linked genes. This overexpression could be related to the clinical features seen in men with KS, including the testicular fibrosis^54–56^. In this study, the only known escapee X-linked DEG found was MXRA5^57^, substantiating its role in KS-related testicular fibrosis. In addition, down-regulation of *TEX11* was found in our second analysis. Mutations of this X-linked gene were previously shown to result in meiotic arrest and azoospermia in infertile men^58^. Furthermore, down-regulation of *C14ORF39* and *SYCE1* were found when comparing KS and TA samples. Variations of these genes have been identified as the reason for infertility in NOA patients^59^.

Winge et al. recently compared all published studies concerning differentially expressed analyses and revealed an overlap of only eight transcripts which were up-regulated in at least two studies on testis tissue, while no down-regulated genes overlapped between the studies^60^. Of these eight genes, only one was found differentially expressed in our analysis of fibrotic versus non-fibrotic tissue, e.g. *DLK1.* This marker for immature Leydig cells has already been shown to be up-regulated in adult men with testicular pathologies, including males with KS^61^. When also including the transcriptomics studies performed on blood samples from men with KS, a total of 139 genes were found to be up- or down-regulated in at least two studies. Of these genes, five could be found in our first analysis (fibrotic vs non-fibrotic): *DLK1*, *RAB34*, *PLSCR2*, *GAPDHS* and *GPA33*. Nevertheless, *GPA33* was found to be up-regulated in our results while it was found down-regulated in the studies by D’Aurora et al. and Belling et al.^15,20^. When comparing the overlapping genes of the transcriptomic studies with our second analysis (KS vs TA), only one overlapping gene was found: *CNGA2*. Nevertheless, this was down-regulated while it was found up-regulated in the study by Winge et al.^21^. The six genes that were found in our analyses and the previously published studies, were also found through the single cell analysis of a KS testis sample by Laurentino et al.^22,60^. Since the other studies compared KS samples with fertile samples, an additional analysis was performed, comparing the differentially expressed genes between the KS and FC samples. In this analysis, 26 of the 139 overlapping genes published in Winge et al. were found^60^. A comparison of the results between the different published papers and our results has been included as Supplementary Information 3—Comparison studies.

In this study, snap-frozen testicular biopsies were used to perform the gene expression analysis, in contrast to most other studies using blood samples or extracted RNA from paraffin-embedded tissue. Nevertheless, this study has a few limitations. Only a small number of samples could be included, as KS samples for research are rather scarce. Since ideal control samples with a fibrotic score of zero were not available, we included samples with fibrotic scores one and two in the non-fibrotic group. A last limitation includes the use of bulk RNA sequencing, which is sensitive to differences in cell-type proportions. Meaning that, each time a differentially expressed gene is reported, it should be taken into account that the differential expression can occur due to cellularity differences between the samples.

## Conclusions

The gene-expression in fibrotic and non-fibrotic testis tissue was compared, revealing genes such as *VCAM1*, which may play a role in testicular fibrosis. In addition, this analysis revealed a pertinent role for genes involved in the TGF-β1 pathway. Secondly, a differential gene expression analysis between the fibrotic groups, KS and TA samples, was performed. An overlap of X-linked genes from our two analyses, revealed the up-regulation of *MXRA5* and *DCX* as well as the down-regulation of *VCX3B.* The altered expression of these genes may lead to mechanisms leading to the KS-related testicular fibrosis. Nevertheless, the function of these genes needs further study.

## Supplementary Information

Supplementary Legends.

Supplementary Information 1.

Supplementary Information 2.

Supplementary Information 3.

Supplementary Figure 1.

Supplementary Figure 2.

Supplementary Figure 3.

Supplementary Figure 4.

Supplementary Figure 5.

Supplementary Figure 6.

Supplementary Figure 7.

Supplementary Figure 8.

Supplementary Figure 9.

Supplementary Table 1.

## Data Availability

The datasets used and analysed during the current study have been deposited in the GEO public database (GSE200680).

## Abbreviations

AFA: Alcohol-formalin acetic acid mixture
DCX: Doublecortin
DEGs: Differentially expressed genes
ECM: Extracellular matrix
FC: Fertile controls
GO: Gene ontology
KEGG: Kyoto encyclopedia of genes and genomes
KS: Klinefelter syndrome
MXRA5: Matrix-remodelling associated 5
RIN: RNA integrity number
SCO: Sertoli cell only syndrome
TA: Mixed testis atrophy
TESE: Testicular sperm extraction
TGF-β1: Transforming growth factor beta-1
VCAM1: Vascular cell adhesion molecule 1
VCX3B: Variable charge X-linked 3B

## References

[1] Klinefelter FH, Reifenstein CE, Albright F (1942). Syndrome characterized by gynecomastia, aspermatogenesis without A-leydigism, and increased excretion of follicle-stimulating hormone. J. Clin. Endocrinol. Metab..

[2] Bojesen A, Juul S, Gravholt CH (2003). Prenatal and postnatal prevalence of Klinefelter syndrome: A national registry study. J. Clin. Endocrinol. Metab..

[3] Lanfranco F, Kamischke A, Zitzmann M, Nieschlag E (2004). Klinefelter's syndrome. Lancet.

[4] Groth KA, Skakkebaek A, Host C, Gravholt CH, Bojesen A (2013). Clinical review: Klinefelter syndrome: A clinical update. J. Clin. Endocrinol. Metab..

[5] Radicioni AF, Ferlin A, Balercia G, Pasquali D, Vignozzi L, Maggi M (2010). Consensus statement on diagnosis and clinical management of Klinefelter syndrome. J. Endocrinol. Invest..

[6] Kanakis GA, Nieschlag E (2018). Klinefelter syndrome: More than hypogonadism. Metab. Clin. Exp..

[7] Bearelly P, Oates R (2019). Recent advances in managing and understanding Klinefelter syndrome. F1000 Res..

[8] Van Saen D, Vloeberghs V, Gies I, Mateizel I, Sermon K, De Schepper J (2018). When does germ cell loss and fibrosis occur in patients with Klinefelter syndrome?. Hum. Reprod..

[9] Aksglaede L, Wikstrom AM, Rajpert-De Meyts E, Dunkel L, Skakkebaek NE, Juul A (2006). Natural history of seminiferous tubule degeneration in Klinefelter syndrome. Hum. Reprod. Update..

[10] Winge SB, Dalgaard MD, Jensen JM, Graem N, Schierup MH, Juul A (2018). Transcriptome profiling of fetal Klinefelter testis tissue reveals a possible involvement of long non-coding RNAs in gonocyte maturation. Hum. Mol. Genet..

[11] Willems M, Gies I, Van Saen D (2020). Germ cell loss in klinefelter syndrome: When and why?. Am. J. Med. Genet. C Semin. Med. Genet..

[12] Corona G, Pizzocaro A, Lanfranco F, Garolla A, Pelliccione F, Vignozzi L (2017). Sperm recovery and ICSI outcomes in Klinefelter syndrome: A systematic review and meta-analysis. Hum. Reprod. Update..

[13] Deebel NA, Galdon G, Zarandi NP, Stogner-Underwood K, Howards S, Lovato J (2020). Age-related presence of spermatogonia in patients with Klinefelter syndrome: A systematic review and meta-analysis. Hum. Reprod. Update..

[14] Zitzmann M, Bongers R, Werler S, Bogdanova N, Wistuba J, Kliesch S (2015). Gene expression patterns in relation to the clinical phenotype in Klinefelter syndrome. J. Clin. Endocrinol. Metab..

[15] Belling K, Russo F, Jensen AB, Dalgaard MD, Westergaard D, Rajpert-De Meyts E (2017). Klinefelter syndrome comorbidities linked to increased X chromosome gene dosage and altered protein interactome activity. Hum. Mol. Genet..

[16] Skakkebaek A, Nielsen MM, Trolle C, Vang S, Hornshoj H, Hedegaard J (2018). DNA hypermethylation and differential gene expression associated with Klinefelter syndrome. Sci. Rep..

[17] Vawter M, Harvey P, DeLisi L (2007). Dysregulation of X-linked gene expression in klinefelter's syndrome and association with verbal cognition. Am. J. Med. Genet. B.

[18] Huang J, Zhang L, Deng H, Chang L, Liu Q, Liu P (2015). Global transcriptome analysis of peripheral blood identifies the most significantly down-regulated genes associated with metabolism regulation in Klinefelter syndrome. Mol. Reprod. Dev..

[19] D'Aurora M, Ferlin A, Di Nicola M, Garolla A, De Toni L, Franchi S (2015). Deregulation of sertoli and leydig cells function in patients with Klinefelter syndrome as evidenced by testis transcriptome analysis. BMC Genomics.

[20] D'Aurora M, Ferlin A, Garolla A, Franchi S, D'Onofrio L, Trubiani O (2017). Testis transcriptome modulation in Klinefelter patients with hypospermatogenesis. Sci. Rep..

[21] Winge SB, Dalgaard MD, Belling KG, Jensen JM, Nielsen JE, Aksglaede L (2018). Transcriptome analysis of the adult human Klinefelter testis and cellularity-matched controls reveals disturbed differentiation of Sertoli- and Leydig cells. Cell Death Dis..

[22] Laurentino S, Heckmann L, Di Persio S, Li X, Meyer ZH, Wistuba J (2019). High-resolution analysis of germ cells from men with sex chromosomal aneuploidies reveals normal transcriptome but impaired imprinting. Clin. Epigenet..

[23] Zhao L, Yao C, Xing X, Jing T, Li P, Zhu Z (2020). Single-cell analysis of developing and azoospermia human testicles reveals central role of Sertoli cells. Nat. Commun..

[24] Mahyari E, Guo J, Lima AC, Lewinsohn DP, Stendahl AM, Vigh-Conrad KA (2021). Comparative single-cell analysis of biopsies clarifies pathogenic mechanisms in Klinefelter syndrome. Am. J. Hum. Genet..

[25] Schindelin J, Arganda-Carreras I, Frise E, Kaynig V, Longair M, Pietzsch T (2012). Fiji: An open-source platform for biological-image analysis. Nat. Methods..

[26] Dobin A, Davis CA, Schlesinger F, Drenkow J, Zaleski C, Jha S (2013). STAR: Ultrafast universal RNA-seq aligner. Bioinformatics.

[27] Anders S, Pyl PT, Huber W (2015). HTSeq: A Python framework to work with high-throughput sequencing data. Bioinformatics.

[28] Love MI, Huber W, Anders S (2014). Moderated estimation of fold change and dispersion for RNA-seq data with DESeq2. Genome Biol..

[29] Kanehisa M, Goto S (2000). KEGG: Kyoto encyclopedia of genes and genomes. Nucleic Acids Res..

[30] Friedel CC, Kaufmann S, Dölken L, Zimmer R (2010). HALO: A Java framework for precise transcript half-life determination. Bioinformatics.

[31] Xu Y, Li J, Liang W, Zhu H (2013). Evaluation on changes of testicular histology in aging men. J. Reprod. Contracept..

[32] Mayerhofer A (2013). Human testicular peritubular cells: more than meets the eye. Reproduction.

[33] Van Saen D, Vloeberghs V, Gies I, De Schepper J, Tournaye H, Goossens E (2020). Characterization of the stem cell niche components within the seminiferous tubules in testicular biopsies of Klinefelter patients. Fertil. Steril..

[34] Willems M, Vloeberghs V, Gies I, De Schepper J, Tournaye H, Goossens E (2020). Testicular immune cells and vasculature in Klinefelter syndrome from childhood up to adulthood. Hum. Reprod..

[35] Mohammadzadeh N, Melleby AO, Palmero S, Sjaastad I, Chakravarti S, Engebretsen KVT (2020). Moderate loss of the extracellular matrix proteoglycan lumican attenuates cardiac fibrosis in mice subjected to pressure overload. Cardiology.

[36] Veräjäkorva E, Laato M, Pöllänen P (2002). CD 99 and CD 106 (VCAM-1) in human testis. Asian J. Androl..

[37] Sainio-Pöllänen S, Sundström J, Erkkilä S, Hänninen A, Vainiopää M, Martikainen M (1997). CD106 (VCAM-1) in testicular immunoregulation. J. Reprod. Immunol..

[38] Abe K, Kon S, Kameyama H, Zhang J, Morohashi KI, Shimamura K (2021). VCAM1-α4β1 integrin interaction mediates interstitial tissue reconstruction in 3-D re-aggregate culture of dissociated prepubertal mouse testicular cells. Sci. Rep..

[39] Agassandian M, Tedrow JR, Sembrat J, Kass DJ, Zhang Y, Goncharova EA (2015). VCAM-1 is a TGF-β1 inducible gene upregulated in idiopathic pulmonary fibrosis. Cell. Signal..

[40] Kong DH, Kim YK, Kim MR, Jang JH, Lee S (2018). Emerging roles of vascular cell adhesion molecule-1 (VCAM-1) in immunological disorders and cancer. Int. J. Mol. Sci..

[41] Ignotz RA, Massagué J (1986). Transforming growth factor-beta stimulates the expression of fibronectin and collagen and their incorporation into the extracellular matrix. J. Biol. Chem..

[42] Gonzalez CR, Matzkin ME, Frungieri MB, Terradas C, Ponzio R, Puigdomenech E (2010). Expression of the TGF-beta1 system in human testicular pathologies. Reprod. Biol. Endocrinol..

[43] Yamaguchi Y, Mann DM, Ruoslahti E (1990). Negative regulation of transforming growth factor-beta by the proteoglycan decorin. Nature.

[44] Ding L, Li S, Zhang Y, Gai J, Kou J (2018). MXRA5 is decreased in preeclampsia and affects trophoblast cell invasion through the MAPK pathway. Mol. Cell. Endocrinol..

[45] Wang GH, Yao L, Xu HW, Tang WT, Fu JH, Hu XF (2013). Identification of MXRA5 as a novel biomarker in colorectal cancer. Oncol. Lett..

[46] Poveda J, Sanz AB, Fernandez-Fernandez B, Carrasco S, Ruiz-Ortega M, Cannata-Ortiz P (2017). MXRA5 is a TGF-β1-regulated human protein with anti-inflammatory and anti-fibrotic properties. J. Cell Mol. Med..

[47] Dijkmans TF, van Hooijdonk LW, Fitzsimons CP, Vreugdenhil E (2010). The doublecortin gene family and disorders of neuronal structure. Cent. Nerv. Syst. Agents Med. Chem..

[48] Zeng L, Gu S, Li Y, Zhao E, Xu J, Ye X (2003). Identification of a novel human doublecortin-domain-containing gene (DCDC1) expressed mainly in testis. J. Hum. Genet..

[49] Fukami M, Kirsch S, Schiller S, Richter A, Benes V, Franco B (2000). A member of a gene family on Xp22.3, VCX-A, is deleted in patients with X-linked nonspecific mental retardation. Am. J. Hum. Genet..

[50] Khalifa MM, Struthers JL (2002). Klinefelter syndrome is a common cause for mental retardation of unknown etiology among prepubertal males. Clin. Genet..

[51] Tuttelmann F, Damm OS, Luetjens CM, Baldi M, Zitzmann M, Kliesch S (2014). Intratesticular testosterone is increased in men with Klinefelter syndrome and may not be released into the bloodstream owing to altered testicular vascularization: A preliminary report. Andrology..

[52] Wistuba J, Beumer C, Warmeling AS, Sandhowe-Klaverkamp R, Stypmann J, Kuhlmann M (2020). Testicular blood supply is altered in the 41, XX Y* Klinefelter syndrome mouse model. Sci. Rep..

[53] Wistuba J, Luetjens CM, Stukenborg JB, Poplinski A, Werler S, Dittmann M (2010). Male 41, XXY* mice as a model for klinefelter syndrome: Hyperactivation of leydig cells. Endocrinology.

[54] Carrel L, Willard HF (2005). X-inactivation profile reveals extensive variability in X-linked gene expression in females. Nature.

[55] Iitsuka Y, Bock A, Nguyen DD, Samango-Sprouse CA, Simpson JL, Bischoff FZ (2001). Evidence of skewed X-chromosome inactivation in 47, XXY and 48, XXYY Klinefelter patients. Am. J. Med. Genet..

[56] Zitzmann M, Depenbusch M, Gromoll J, Nieschlag E (2004). X-chromosome inactivation patterns and androgen receptor functionality influence phenotype and social characteristics as well as pharmacogenetics of testosterone therapy in Klinefelter patients. J. Clin. Endocrinol. Metab..

[57] WainerKatsir K, Linial M (2019). Human genes escaping X-inactivation revealed by single cell expression data. BMC Genom..

[58] Yatsenko AN, Georgiadis AP, Röpke A, Berman AJ, Jaffe T, Olszewska M (2015). X-linked TEX11 mutations, meiotic arrest, and azoospermia in infertile men. N. Engl. J. Med..

[59] Hou D, Yao C, Xu B, Luo W, Ke H, Li Z (2022). Variations of C14ORF39 and SYCE1 identified in idiopathic premature ovarian insufficiency and nonobstructive azoospermia. J. Clin. Endocrinol. Metab..

[60] Winge SB, Soraggi S, Schierup MH, Rajpert-De Meyts E, Almstrup K (2020). Integration and reanalysis of transcriptomics and methylomics data derived from blood and testis tissue of men with 47, XXY Klinefelter syndrome indicates the primary involvement of Sertoli cells in the testicular pathogenesis. Am. J. Med. Genet. C..

[61] Lottrup G, Nielsen JE, Maroun LL, Moller LM, Yassin M, Leffers H (2014). Expression patterns of DLK1 and INSL3 identify stages of Leydig cell differentiation during normal development and in testicular pathologies, including testicular cancer and Klinefelter syndrome. Hum. Reprod..

