# Supplementary material for: Transcriptomic differences between fibrotic and non-fibrotic testicular tissue reveal possible key players in Klinefelter syndrome-related testicular fibrosis
Source: Sci Rep. 2022 Dec 13;12:21518. doi: 10.1038/s41598-022-26011-6 (PMC9748020; doi:10.1038/s41598-022-26011-6)
Supplement: Supplementary file 1 — Supplementary Legends. [file 41598_2022_26011_MOESM1_ESM.docx]

Supplementary figure 1: H/PAS analysis of all samples included in the RNA sequencing analysis.

Supplementary figure 2: PCA analysis of the fibrotic versus non-fibrotic RNA seq analysis.

Supplementary figure 3: Age of patients included in the fibrotic group compared to those of the non-fibrotic group. No statistical difference was found.

Supplementary figure 4: Enrichment analysis of the fibrotic versus non-fibrotic analysis. Gene ontology analysis of the biological functions of the down-regulated genes.

Supplementary figure 5: RT-qPCR analysis for TGF-β1 in KS, SCO and FC testicular samples. A significant difference was found between KS and FC samples. *p=0.0016

Supplementary figure 6: Overview of all DEGs found per chromosome. **A)** DEGs found in the first analysis, comparing fibrotic and non-fibrotic testis tissue. **B)** DEGs found in the KS versus TA analysis.

Supplementary figure 7: Gene ontology analysis of the biological functions of the down-regulated genes found in the analysis comparing KS and TA samples.

Supplementary figure 8: KEGG analysis found in the analysis comparing KS and TA samples. **A)** Up-regulated DEGs. **B)** Down-regulated DEGs.

Supplementary figure 9: Quantification of the RNA *in situ* hybridization validation. The graph shows the percentage of cells within the KS, FC, TA and SCO samples which express *VCAM1, MXRA5* and *DCX.* With a clear increase of expression of all genes in the KS tissue. In addition, the quantification of a KS sample for the *VCAM1/MXRA5* duplex with the HALO software is shown.
