# Supplementary material for: Transcriptomic differences between fibrotic and non-fibrotic testicular tissue reveal possible key players in Klinefelter syndrome-related testicular fibrosis
Source: Sci Rep. 2022 Dec 13;12:21518. doi: 10.1038/s41598-022-26011-6 (PMC9748020; doi:10.1038/s41598-022-26011-6)
Supplement: Supplementary file 4 — Supplementary Information 3. [file 41598_2022_26011_MOESM4_ESM.docx]

Supplemental experimental procedure

**Patient samples**

Adult KS (n=5), SCO (n=5) and TA (n=5) testicular tissue samples used for quantitative RT-PCR were collected from the pathology department of the UZ Brussel from patients undergoing a TESE procedure as part of their fertility treatment. Control testicular tissues from healthy fertile men (n=5) were obtained from patients undergoing a vasectomy reversal. Immediately after the biopsy procedure samples were snap frozen in liquid nitrogen and stored at -80°C until further processing in the BITE lab.

**Quantitative real time PCR**

RNA was extracted from adult testicular tissue using the RNeasy Mini Kit (74104; Qiagen, Antwerp, Belgium) according to the manufacturer’s instructions. RNA was reverse transcribed into cDNA with NotI(dT) primers using the First-Strand cDNA Synthesis Kit (27926101; GE Healthcare life sciences, Diegem, Belgium) according to the guidelines supplied with the kit. cDNA concentration was measured using the Nanodrop spectrophotometer (Isogen Life Science, De Meern, Netherlands). The quantitative RT-PCR reaction was performed with the ViAA7 thermocycler and ViAA7 software (Termo Fischer Scientific, Merelbeke, Belgium). For each reaction, 20 μl of final volume was used, including 40 ng of cDNA, Taqman® Fast advanced Master Mix (2020-03-31; Thermo Fischer Scientific), TaqMan® gene-expression assays for the TGF-β (Hs00998133_m1) and housekeeping genes glyceraldehyde-3-phosphate dehydrogenase 9 (Hs02786624_g1) and glucuronidase β (Hs99999908_m1). For each gene, triplicates were tested per patient. Cycling parameters of the reaction consisted of 50°C for two minutes, 95°C for 10 minutes and 40 cycles of 95°C for 15 seconds and 60°C for one minute.

**Statistical analysis**

Statistical analysis was performed by using GraphPad Prism 8 software. A one-way anova test (Kruskal Wallis test) with Dunn’s multiple comparison was used to determine a statistical difference between multiple variables. Data are presented as dot plots with general mean. P-values <0.05 were considered

significant.
