# Supplementary figures and images for: Transcriptomic differences between fibrotic and non-fibrotic testicular tissue reveal possible key players in Klinefelter syndrome-related testicular fibrosis
Source: Sci Rep. 2022 Dec 13;12:21518. doi: 10.1038/s41598-022-26011-6 (PMC9748020; doi:10.1038/s41598-022-26011-6)

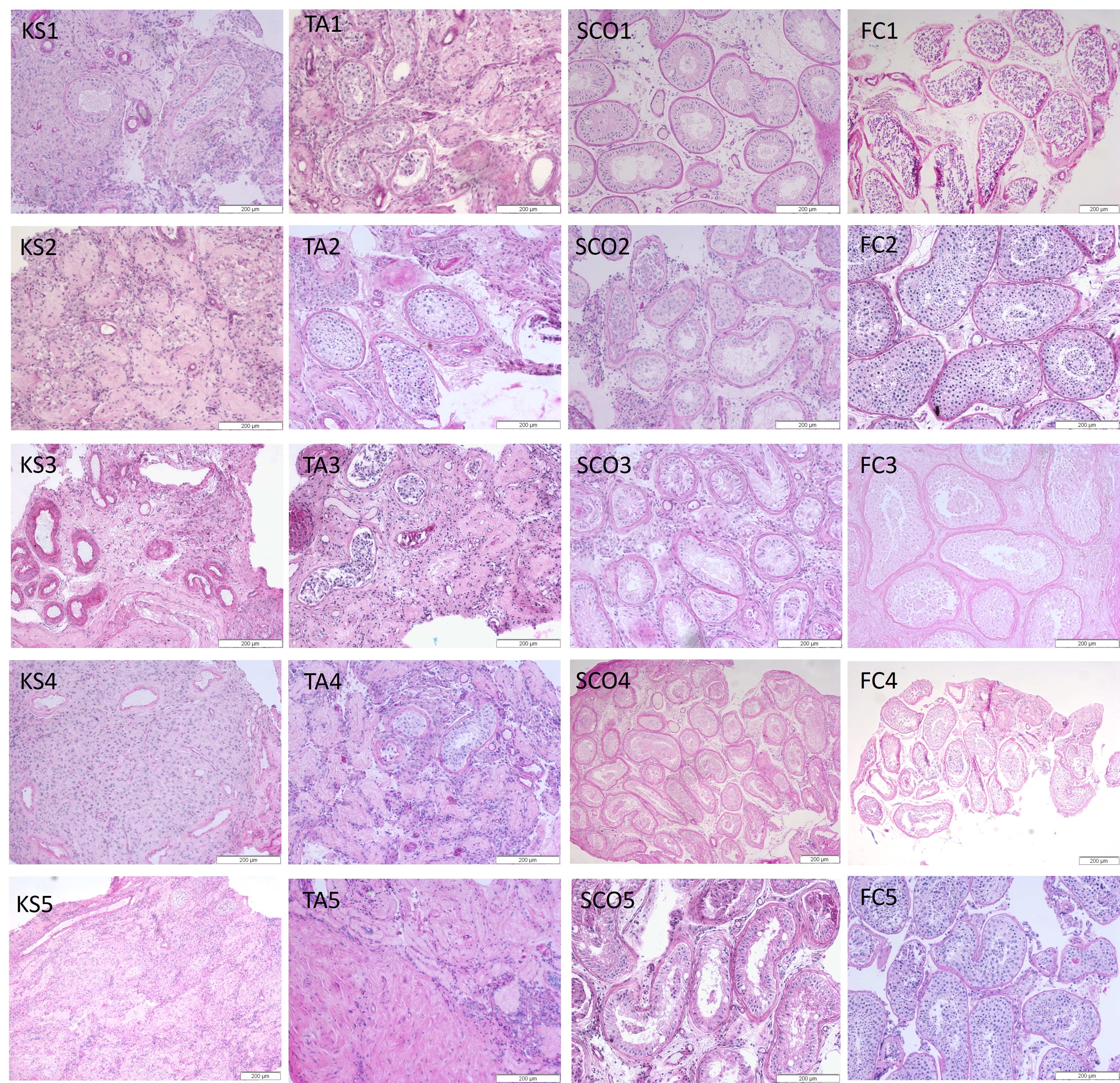

Supplement: Supplementary file 5 — Supplementary Figure 1. [file 41598_2022_26011_MOESM5_ESM.jpg]

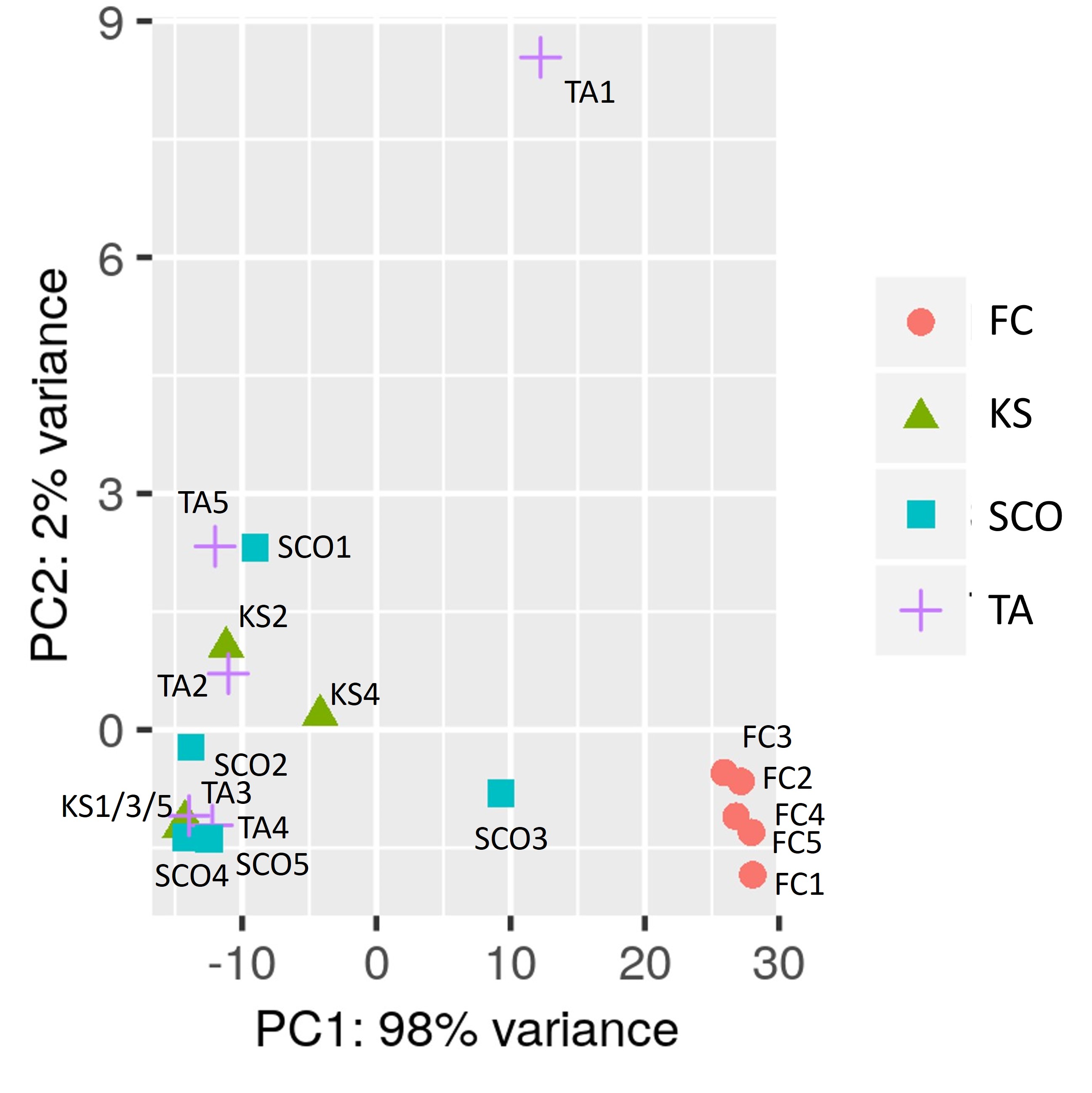

Supplement: Supplementary file 6 — Supplementary Figure 2. [file 41598_2022_26011_MOESM6_ESM.jpg]

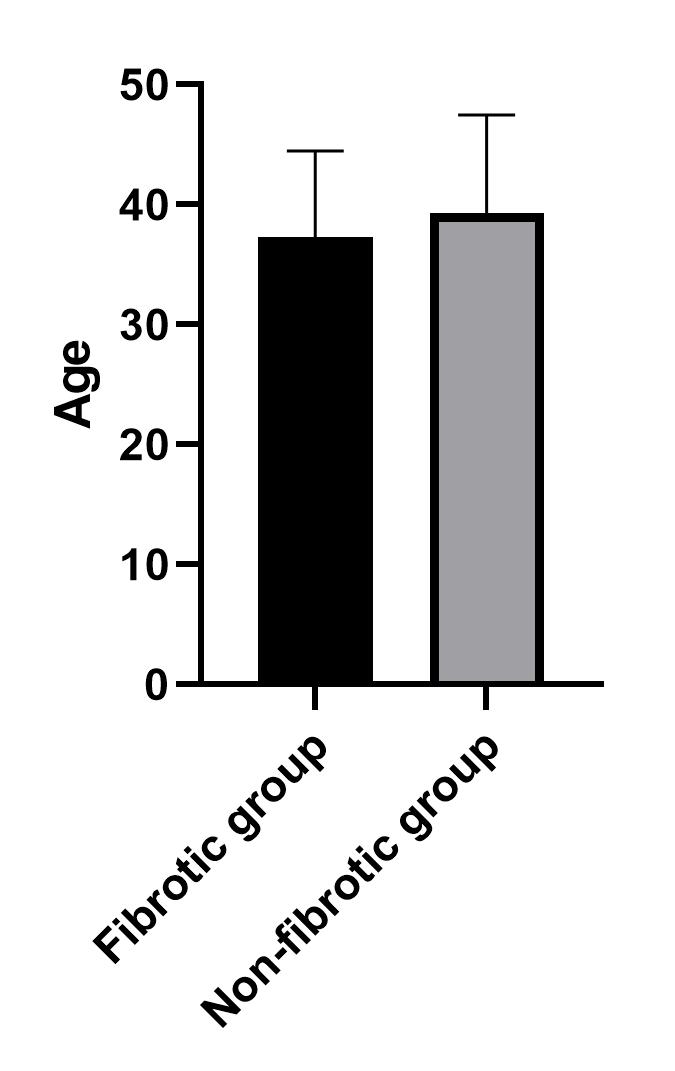

Supplement: Supplementary file 7 — Supplementary Figure 3. [file 41598_2022_26011_MOESM7_ESM.jpg]

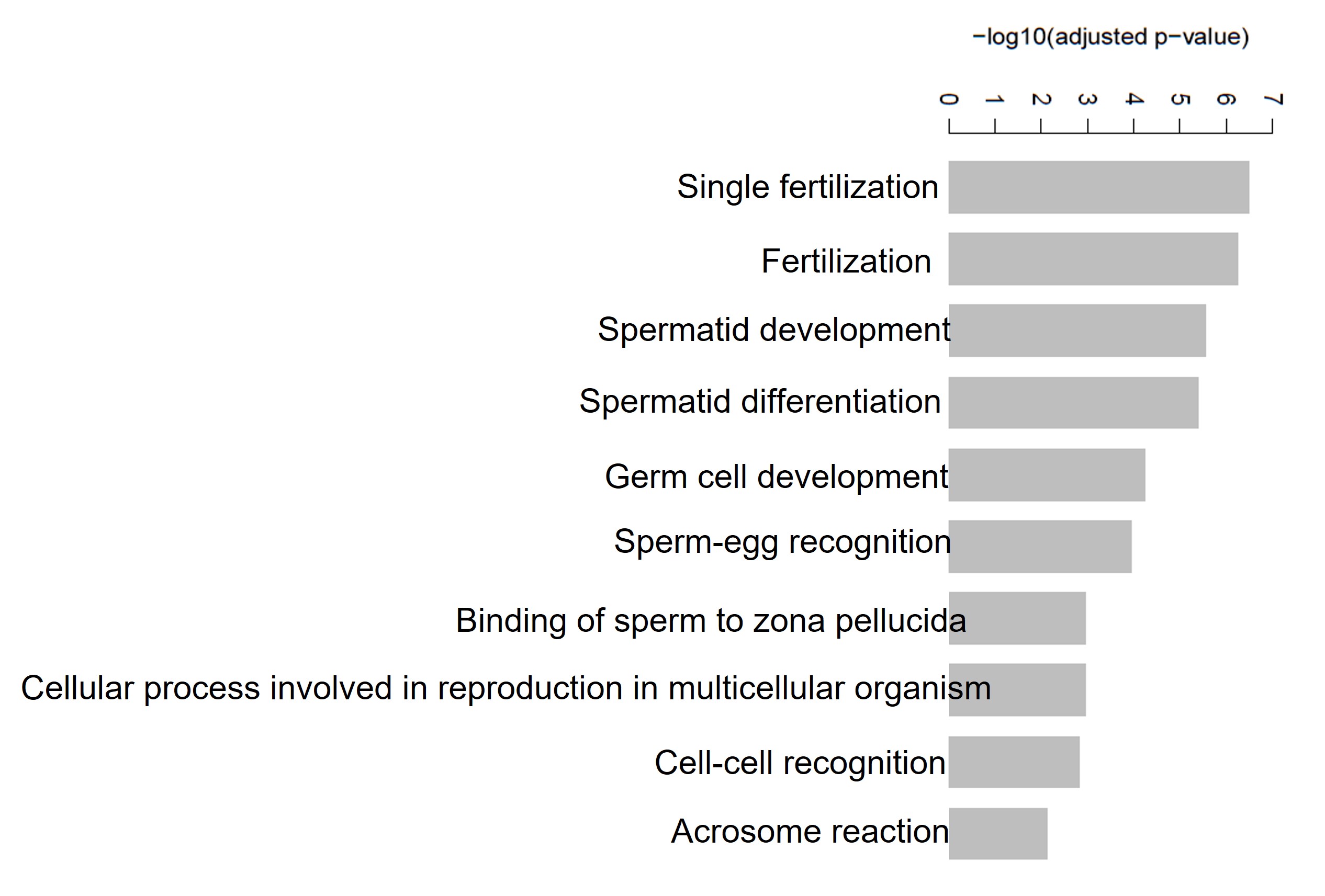

Supplement: Supplementary file 8 — Supplementary Figure 4. [file 41598_2022_26011_MOESM8_ESM.jpg]

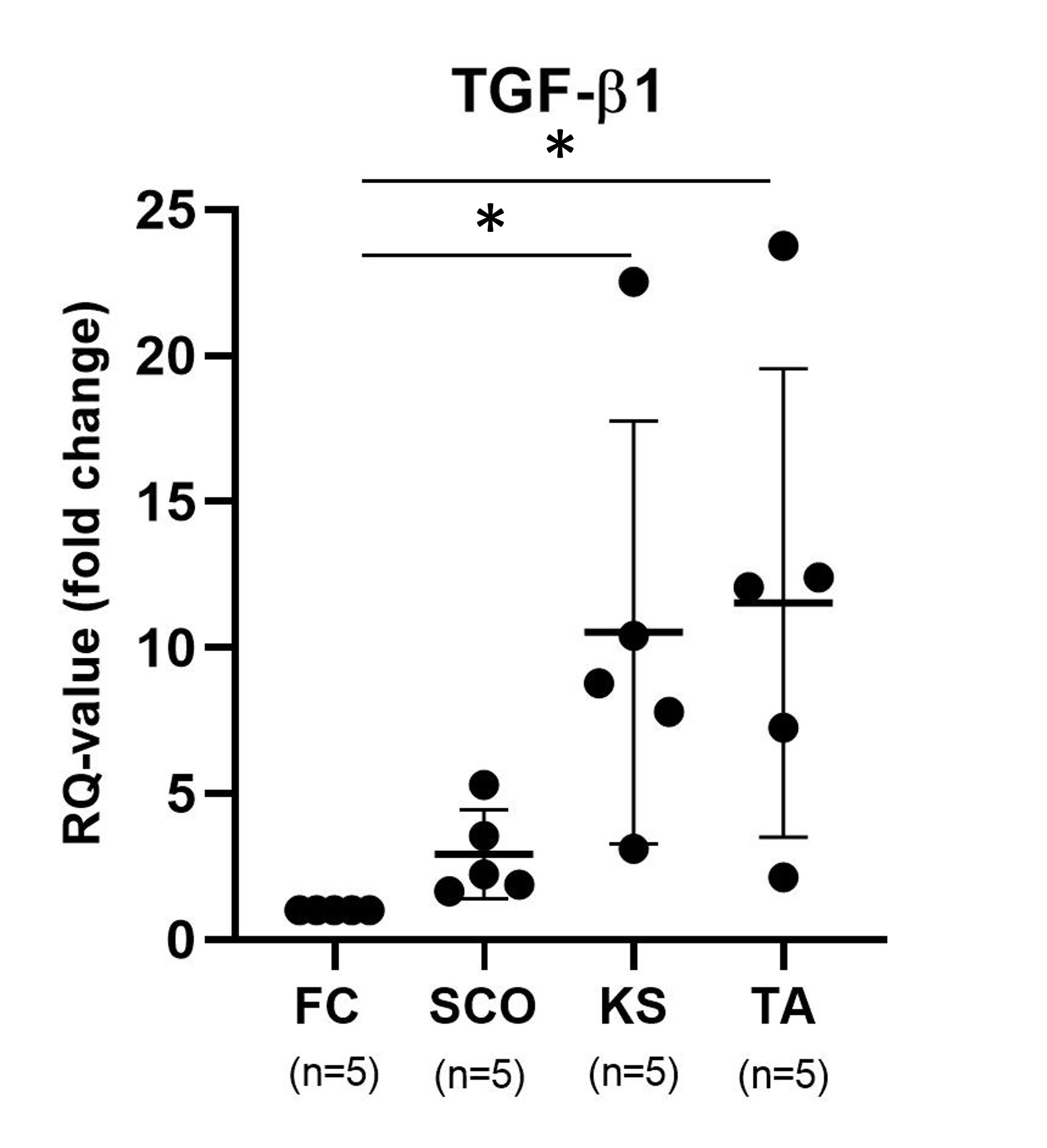

Supplement: Supplementary file 9 — Supplementary Figure 5. [file 41598_2022_26011_MOESM9_ESM.jpg]

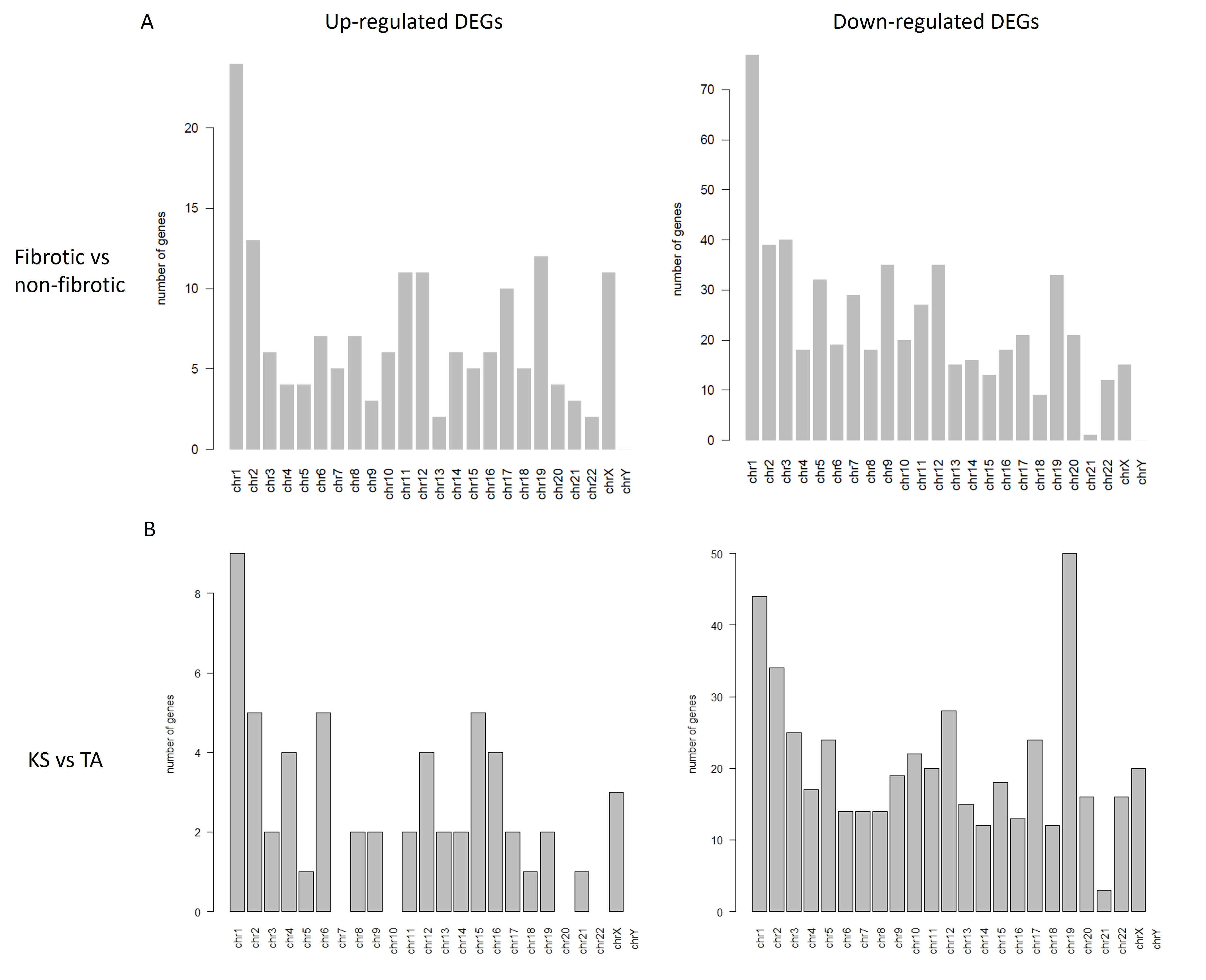

Supplement: Supplementary file 10 — Supplementary Figure 6. [file 41598_2022_26011_MOESM10_ESM.jpg]

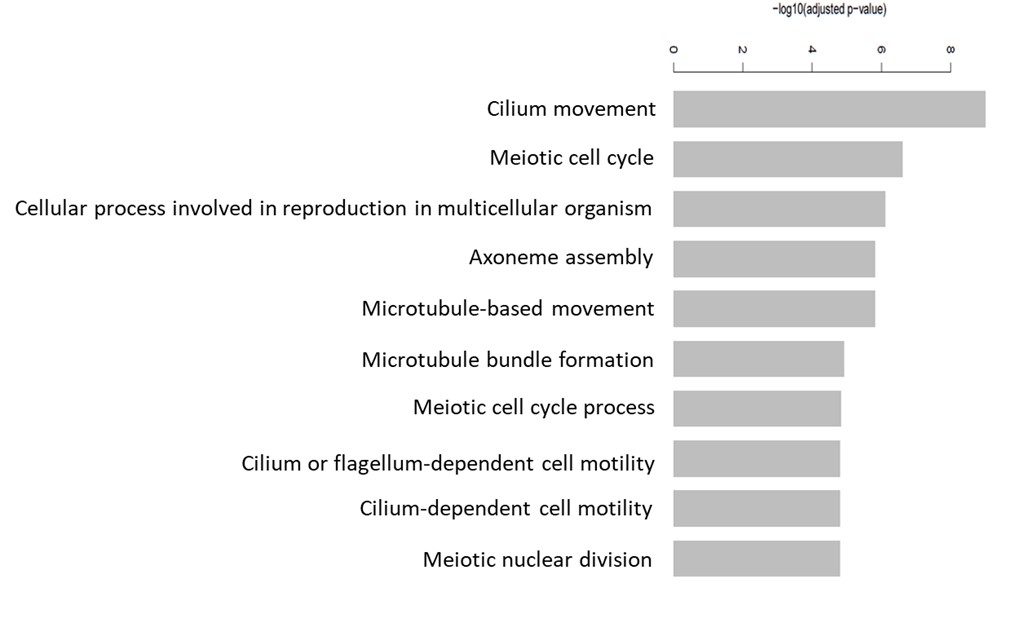

Supplement: Supplementary file 11 — Supplementary Figure 7. [file 41598_2022_26011_MOESM11_ESM.jpg]

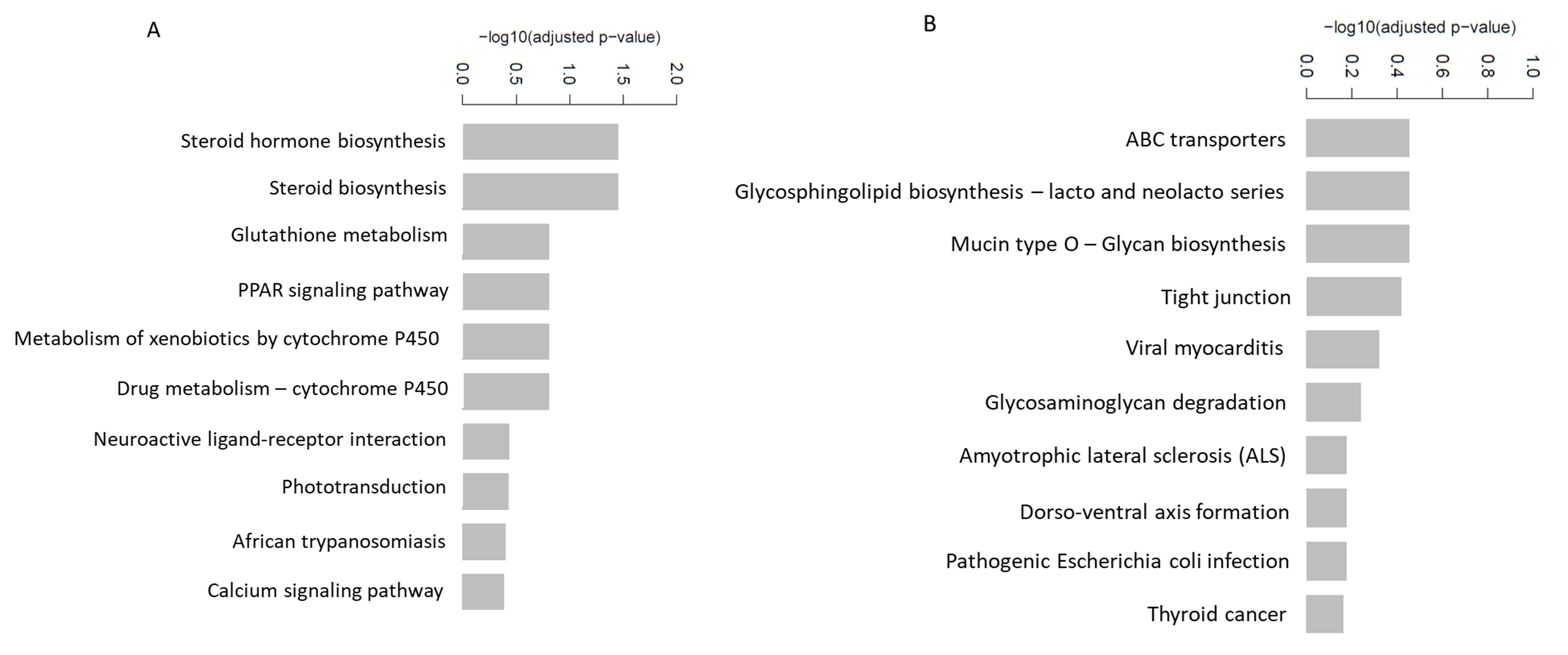

Supplement: Supplementary file 12 — Supplementary Figure 8. [file 41598_2022_26011_MOESM12_ESM.jpg]

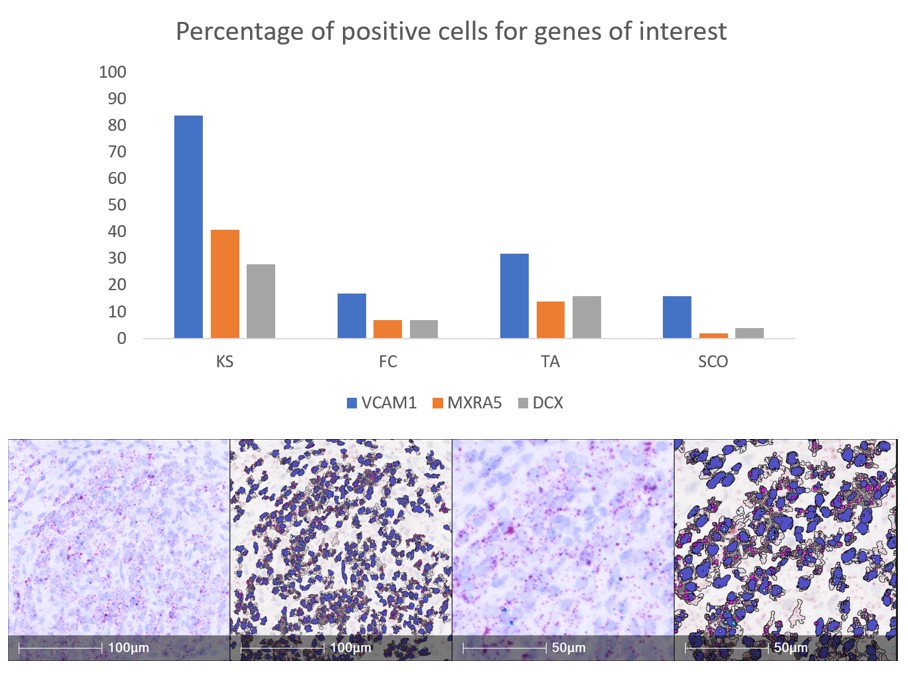

Supplement: Supplementary file 13 — Supplementary Figure 9. [file 41598_2022_26011_MOESM13_ESM.jpg]
