# Supplementary material for: Transcriptomic differences between fibrotic and non-fibrotic testicular tissue reveal possible key players in Klinefelter syndrome-related testicular fibrosis
Source: Sci Rep. 2022 Dec 13;12:21518. doi: 10.1038/s41598-022-26011-6 (PMC9748020; doi:10.1038/s41598-022-26011-6)
Supplement: Supplementary file 14 — Supplementary Table 1. [file 41598_2022_26011_MOESM14_ESM.docx]

Supplementary table I: RIN score and sequencing stats for the included samples

| Patient ID | RIN score | Number of reads | Percentage aligned reads |
| --- | --- | --- | --- |
| KS1 | 8.8 | 40254165 | 72,43 |
| KS2 | 8.3 | 38754423 | 76,7 |
| KS3 | 7.8 | 39444871 | 80,85 |
| KS4 | 7.4 | 31488353 | 79,61 |
| KS5 | 8.8 | 35101366 | 80,5 |
| TA1 | 6.5 | 39793973 | 82,86 |
| TA2 | 5.8 | 43886633 | 79,28 |
| TA3 | 7.3 | 41397316 | 36,96 |
| TA4 | 7.9 | 38721527 | 72,82 |
| TA5 | 7.4 | 43784740 | 84,13 |
| SCO1 | 5.3 | 33031271 | 80,12 |
| SCO2 | 8.4 | 34803193 | 82,39 |
| SCO3 | 7.2 | 42361318 | 75,24 |
| SCO4 | 7.1 | 36047676 | 80,04 |
| SCO5 | 4.7 | 36670089 | 77,99 |
| FC1 | 7 | 42190473 | 82,95 |
| FC2 | 7.6 | 43314658 | 83,65 |
| FC3 | 7.4 | 36150249 | 82,53 |
| FC4 | 7.8 | 32852033 | 81,48 |
| FC5 | 6.6 | 32786700 | 69,44 |
